# Association of body mass index trajectories with incidence of stroke among elderly Chinese adults: a 10-year cohort study

**DOI:** 10.3389/fnut.2026.1804187

**Published:** 2026-06-10

**Authors:** Xiaoyu Zhang, Yuqian Zhang, Wei Li, Yaxuan Zhou, Liang Wang, Mei Yang, Yan Guo

**Affiliations:** 1 School of Public Health, Wuhan University of Science and Technology, Wuhan, Hubei, China; 2 Research Center for Health Promotion in Women, Youth and Children, Wuhan University of Science and Technology, Wuhan, Hubei, China; 3 Department of Pharmacy, Maternal and Child Health Hospital of Hubei Province, Wuhan, Hubei, China; 4 Wuhan Centers for Disease Prevention and Control, Wuhan, Hubei, China

**Keywords:** cohort study, elderly individuals, group-based trajectory modelling, obesity, stroke, trajectory

## Abstract

**Background:**

Limited data exists on relationship between body mass index (BMI) trajectory and stroke risk among elderly Chinese individuals. This study aimed to examine the association of BMI trajectory with stroke among people aged 65 years and older in China.

**Methods:**

150, 813 participants with at least three BMI measurements from 2012 to 2022 were included. Group-based trajectory modeling was used to identify BMI trajectories. Cox proportional hazards regression models were used to examine the association between BMI trajectories and incidence of stroke.

**Results:**

We identified 3 (stable, slowly increased, greatly increased), 4 (slightly decreased, stable, slowly increased, sharply increased), 4 (large decreased, slightly decreased, stable, moderate increased) and 4 (substantially decreased, slightly decreased, stable, slightly increased) BMI trajectories in groups with underweight, normal weight, overweight and obesity at baseline, respectively. Taking the stable weight subgroup as the reference group, among subjects with normal weight, the adjusted hazard ratios (HRs) [95% confidence interval (CI)] were 0.93 (0.88, 0.99) for slightly decreased group, 1.07 (1.03, 1.13) for slowly increased group, and 1.12 (1.02, 1.23) for sharply increased group. Among participants with overweight, the HRs (95% CI) were 0.84 (0.75, 0.95) for large decreased group and 0.94 (0.89, 0.99) for slightly decreased group. Among participants with obesity, the HRs (95% CI) were 0.79 (0.64, 0.96) for large decreased group.

**Conclusion:**

Among people aged 65 years and older, increased BMI was associated with a higher risk of stroke, while decreased BMI was related to reduced risk of stroke.

## 1 Introduction

Stroke is a neurological deficit attributed to an acute focal injury of the central nervous system by a vascular cause, including ischemic stroke (IS), intracerebral hemorrhage (ICH), and subarachnoid hemorrhage (SAH). Common symptoms include hemiparesis, dysarthria, sensory deficits, aphasia, and visual deficits (1–3). Over the past three decades, the absolute number of incidents, deaths and disability adjusted life years (DALYs) due to stroke increased by 70.0, 43.0, and 32.0%, respectively, (4, 5). Especially among the elderly aged 65 and older, it is estimated that the incidence, prevalence, deaths, and DALYs will increase to 75.98, 81.76, 94.41, and 84.45% by 2050 (6, 7). The majority of strokes occur in individuals over 65 years old (8, 9), with over half of survivors experiencing diminished mobility (9). As the second leading cause of both disability and death, stroke poses a staggering burden on the elderly (4, 5, 10, 11). Given that stroke is preventable to a significant extent, it is essential to identify and improve modifiable risk factors to reduce the burden of stroke disease and improve the health of the elderly (4, 12, 13).

Body Mass Index (BMI), defined as body weight in kg divided by height in meters squared, is the metric to define anthropometric obesity characteristics in adults at present. Epidemiological studies have proved that high BMI is one of the five risk factors for stroke (14–16). Previous studies have predominantly focused on the association between single-time point BMI measurements and stroke risk. Mechanistically, obesity increases the incidence of cardiovascular disease primarily through adaptive changes in the structure and function of the cardiovascular system, which occur in response to elevated body weight (17). More importantly, BMI is a dynamic indicator that fluctuates throughout an individual’s lifespan, and its longitudinal trajectory may have a significant impact on health outcomes. Despite two prior studies demonstrated a positive association between changes in BMI trajectory and lifetime stroke risk, one study tracked the BMI changes of adults for only 4 years, which may have restricted the magnitude of observable fluctuations in BMI trajectories (18), and the other study was conducted within a European population, potentially limiting the generalizability of its findings to other ethnic groups (19), the association between variation in BMI trajectory and stroke risk among Chinese elderly warrants further investigation.

Group-Based Trajectory Modelling (GBTM) is a semi-parametric model for longitudinal data that describes the course of an outcome over age or time and identifies several unobservable potential categories in the population according to the variation trend of exposures (20–23). Applying this model, we conducted a 10-year longitudinal study targeting at Chinese elderly individuals on the relationship between BMI trajectory and the risk of stroke.

## 2 Materials and methods

### 2.1 Study population

**Figure 1:**
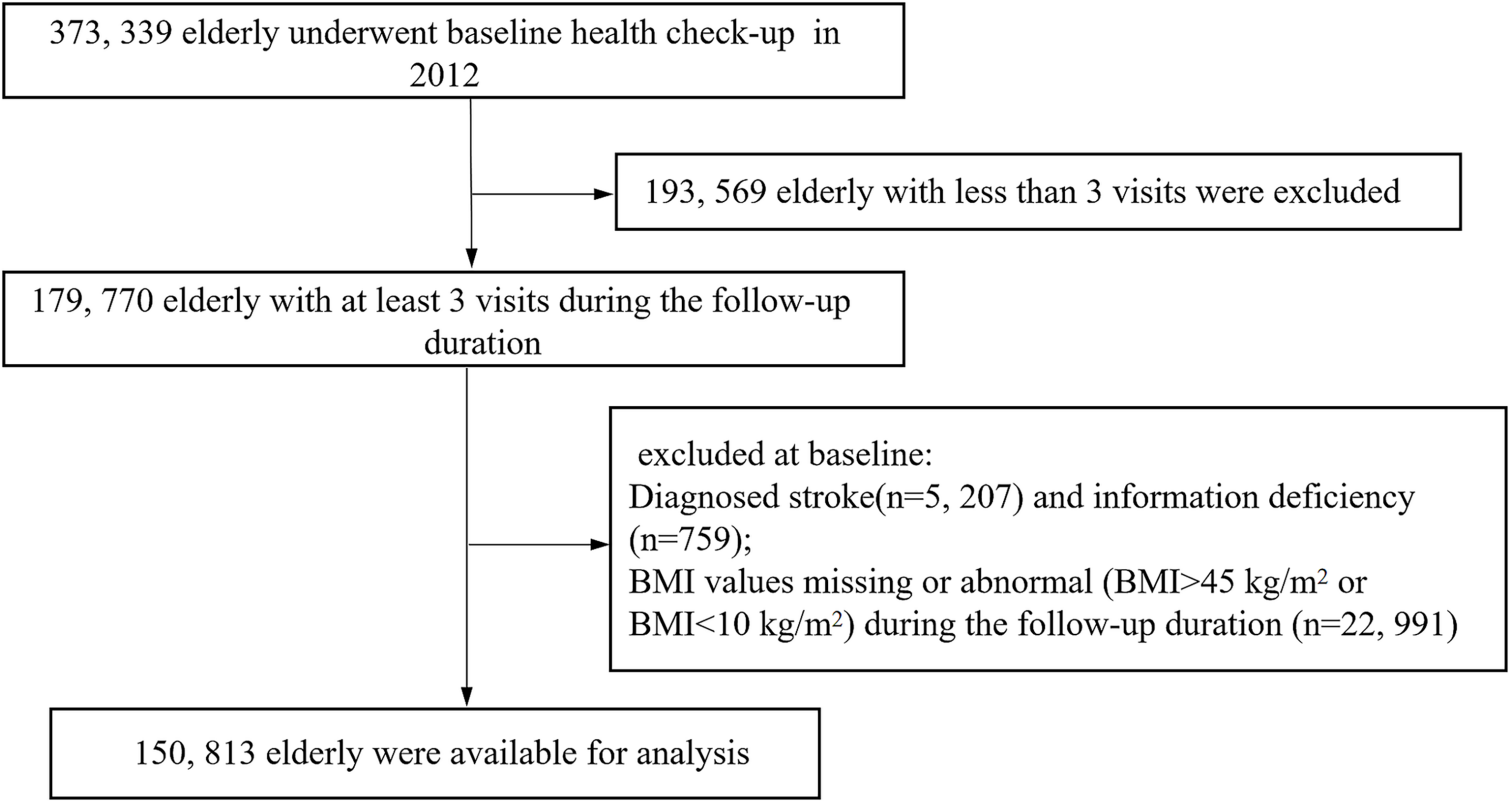
Flow chart of data screening.

The participants of this study were from the Elderly Health Management Information System in Wuhan City, Hubei Province, central China. The baseline study was carried out in 2012 with the enrollment of 373, 339 people aged 65 years and older. The inclusion criteria comprised individuals aged ≥65 years who were registered residents of Wuhan. Follow-up examinations were conducted in 2014, 2016, 2018, 2019, 2020, 2021, and 2022, giving a total follow-up time of 10 years. Individuals who had any of the following conditions were excluded: less than 3 times follow-up visits, a history of stroke at baseline. Individuals with extreme or wrong values (BMI > 45 kg/m^2^ or BMI < 10 kg/m^2^) in BMI were also excluded. After excluding, 150, 813 participants were included as the final analytic sample (Figure 1).

### 2.2 Measurements

The investigation in this study consisted of two main components: a questionnaire survey and physical examinations. The examination and information were collected by trained medical personnel from community medical institutions. The questionnaire survey collected detailed information on demographic characteristics, lifestyle factors, medical history, and other basic information. The physical examination, which included height and weight, was performed after at least 8 h fasting through field measurements. Participants were measured using standardized protocols, without shoes and in lightweight clothing. Laboratory tests included blood glycemia and lipids. Serum glucose was determined by glucose oxidase method. Triglyceride (TG) and total cholesterol (TC) were measured with enzymatic method.

### 2.3 Follow-up and outcomes

The participants received the questionnaire and physical examination every 2 years between 2012 and 2018 and every year between 2019 and 2022. The outcome event in this study was defined as the occurrence of a first stroke, and the end point was the onset of stroke or the end of the follow-up period. In this study, participants who did not experience a stroke during the preceding round of investigation but had a stroke detected in the subsequent survey round were identified as having a new stroke event. Survival time was defined as time between baseline and the date of newly diagnosed stroke, censoring due to missing data, death, or end of follow-up, whichever came first. Participants who met one of the following criteria were diagnosed with stroke: (1) self-reported having been diagnosed with stroke by a physician; (2) currently taking stroke medication.

### 2.4 Covariate assessment

Information on covariates was obtained through questionnaires. Covariates adjusted in this study included age, sex, marital status, educational level, physical exercise, smoking status, drinking status, TG, TC, and disease status of hypertension, diabetes, hyperlipidemia, coronary heart disease and family history of stroke. Marital status was defined as married and non-married (single, divorced, or widowed). Educational level was categorized into 0–6 years, 7–12 years and >12 years. Smoking status was defined as current smoking and non-current smoking (never or quitting smoking). Drinking status was defined as current drinking and non-current drinking (never or quitting drinking). Exercise was divided into regular physical exercise (moderate-intensity exercise more than three times per week, for at least 30 min per session) or not. Diabetes was defined as having at least one of the following conditions: fasting blood glucose ≥7.0 mmol/L, self-reported, physician diagnoses, or having glucose-lowering medications. Hypertension was defined as having at least one of the following conditions: measured systolic blood pressure ≥140 mm Hg or diastolic blood pressure ≥90 mm Hg, self-reported, physician diagnoses, or having anti-hypertension drugs. Hyperlipidemia was defined as self-reported or physician diagnoses. Coronary heart disease was defined as self-reported or physician diagnoses. Family history of stroke was defined as self-reported.

### 2.5 Statistical analysis

We conducted GBTM to identify distinct groups within a larger population that share similar developmental trajectories over time. The SAS “Traj” plugin was used to display age-scaled BMI trajectories from 2012 to 2022. In accordance with the Chinese Obesity Diagnosis and Treatment Guidelines (2024) issued by the National Health Commission of the People’s Republic of China among Chinese adults, the classification of BMI among Chinese adults at baseline is as follows: underweight was defined as BMI < 18.5 kg/m^2^, normal weight as 18.5 kg/m^2^ ≤ BMI < 24 kg/ m^2^, overweight as 24 kg/ m^2^ ≤ BMI < 28 kg/ m^2^, and obesity as BMI ≥ 28 kg/ m^2^. Based on the criteria, participants were initially categorized into 4 groups. Subsequently, a possible of 2 to 7 trajectories for each group were tested to find the best number of trajectories in each group. Since BMI is regarded as a continuous variable, we chose a censored normal model to fit the trajectories. Trajectories of different shapes (linear, quadratic, or cubic) were used for fitting. The selection of the best-fitting model was based on the Bayesian Information Criterion (BIC), with the following criteria applied: (1) A lower BIC value; (2) The average of the posterior probabilities of group membership exceeding 0.7; and (3) A group sample size greater than 5%. Ultimately, 15 distinct BMI trajectories were identified as the best fit for the data (Supplementary Table 1).

Continuous variables were presented as means with standard deviation. Categorical variables were expressed as frequency and percentage. Means and proportions of population characteristics were compared using Student’s t test for continuous variables and Pearson’s *χ^2^* for categorical variables, respectively. The association of BMI trajectory with stroke was assessed using cox regression models and presented as hazard ratio (HR) and 95% confidence interval (CI). The adjustments were accomplished via 3 models: Model 1 was unadjusted; Model 2 was adjusted for age and sex; Mode 3 was additionally adjusted for marital status, physical exercise, smoking status, drinking status, TG, TC, diabetes, hypertension, hyperlipidemia, coronary heart disease and family history of stroke. In addition, the stratified analyses were conducted in the present study according to sex and the age groups (65–70 years, 70–75 years, 75–80 years, and ≥ 80 years).

All statistical analyses were performed using SAS software version 9.4 (SAS Institute, Cary, NC). Two-sided *p*-values < 0.05 were considered statistically significant.

## 3 Results

### 3.1 Baseline characteristics of the subjects

This study included a total of 150, 813 participants, among whom the mean age was 71.36 years (SD 4.61) and 53.87% were women. The incidence of stroke during 2012 to 2022 in the study population was 12.41%. As shown in Table 1, participants who experienced a new stroke event exhibited significantly higher BMI, levels of fasting plasma glucose (FPG), TG, and TC, as well as elevated rates of diabetes, hypertension and hyperlipidemia, compared with those who did not experience a stroke (*p*-values < 0.05).

**Table 1:** Characteristics of 150,813 study participants at baseline.

Characteristics	Total (*N* = 150,813)	Non-stroke (*N* = 132,099)	Stroke (*N* = 18,714)	*p* value
Age, years, mean (SD)	71.36 (4.61)	71.40 (4.65)	71.06 (4.26)	<0.0001
Sex, *n* (%)				<0.0001
Male	69,571 (46.13)	61,231 (46.35)	8,340 (44.57)	
Female	81,242 (53.87)	70,868 (53.65)	10,374 (55.43)	
BMI	23.32 (3.31)	23.28 (3.31)	23.62 (3.27)	0.007
Educational level, *n* (%)				<0.0001
No formal education	85,409 (56.63)	74,926 (56.72)	10,681 (57.07)	
1–6 years	45,423 (30.12)	39,426 (29.85)	5,799 (30.99)	
7–12 years	14,429 (9.57)	12,769 (9.67)	1,660 (8.87)	
>12 years	5,552 (3.68)	4,978 (3.77)	574 (3.07)	
Marital status, *n* (%)				0.000
Married	115,988 (76.91)	101,387 (76.75)	14,601 (78.02)	
Non-married	34,825 (23.09)	30,712 (23.25)	4,113 (21.98)	
Regular exercise, *n* (%)	43,104 (28.58)	37,579 (28.45)	5,525 (29.52)	0.002
Smoking status, *n* (%)				0.004
Non-smoking	114,093 (75.65)	99,777 (75.53)	14,316 (76.50)	
Current smoking	34,872 (23.12)	30,713 (23.25)	4,159 (22.22)	
Missing	1848 (1.23)	1,609 (1.22)	239 (1.28)	
Drinking status, *n* (%)				<0.0001
Non-alcohol drinking	112,626 (74.68)	98,383 (74.48)	14,243 (76.11)	
Current alcohol drinking	36,757 (24.37)	32,469 (24.58)	4,288 (22.91)	
Missing	1,430 (0.95)	1,247 (0.94)	183 (0.98)	
FPG, mean (SD)	5.45 (1.38)	5.44 (1.37)	5.47 (1.38)	0.325
TG, mean (SD)	1.39 (0.91)	1.39 (0.91)	1.44 (0.93)	0.003
TC, mean (SD)	4.91 (1.00)	4.91 (1.00)	4.96 (1.00)	0.274
Diabetes, *n* (%)	10,454 (6.93)	8,894 (5.59)	1,560 (8.33)	<0.0001
Hypertension, *n* (%)	50,439 (33.44)	42,378 (32.08)	8,061 (43.07)	<0.0001
Hyperlipidemia, *n* (%)	8,864 (5.88)	7,386 (5.66)	1,478 (7.90)	<0.0001

BMI, body mass index; FPG, fasting plasma glucose; TG, triglyceride; TC, total cholesterol; SD, standard deviation. Participants were grouped according to incident stroke events during the follow-up period. None of the participants had experienced a stroke at baseline.

### 3.2 BMI trajectory groups

**Figure 2:**
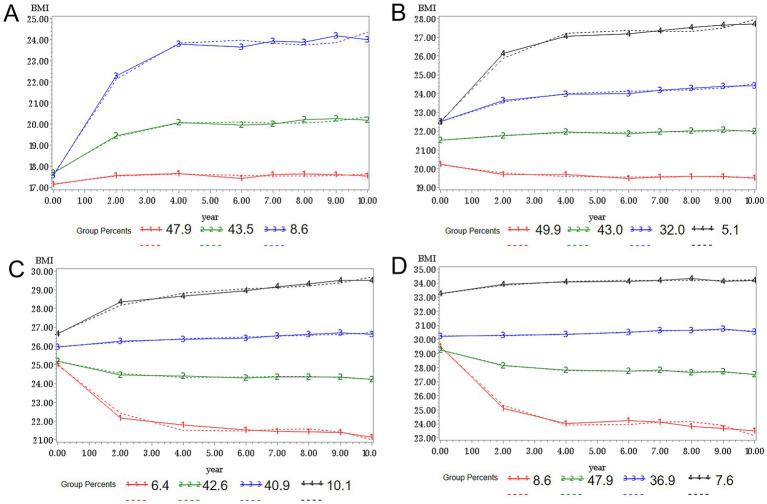
BMI trajectories groups from 2012 to 2022. **(A)** Trajectories of group with underweight; **(B)** trajectories of group with normal weight; **(C)** trajectories of group with overweight; **(D)** trajectories of group with obesity.

GBTM identified 15 trajectory groups over the 10-year follow-up period overall. As illustrated in Figure 2, participants were categorized into 4 groups based on BMI at baseline: underweight, normal weight, overweight, and obese. The changes in BMI values are now categorized into the following categories based on the magnitude of the fluctuations: stable, slight, moderate, and significant. Among participants with underweight, 3 trajectories were identified: stable, slightly increased, and large increased. Among subjects with normal-weight, BMI changes were characterized by four 4 patterns: slightly decreased, stable, slightly increased, and large increased. Among participants with overweight, we observed 4 trajectories: large decreased, slightly decreased, stable, and moderate increased. Finally, the analysis revealed the presence of 4 distinct categories among subjects with obesity: large decreased, slightly decreased, stable, and slightly increased.

### 3.3 The relationship between BMI trajectories and stroke risks

As shown in Table 2, the association in BMI and stroke was not significant in group with underweight. After fully adjustment, taking the participants in stable weight group as the reference group, among subjects with normal weight, those in the slightly decreased weight group had a reduced risk of stroke (HR: 0.93; 95%CI: 0.88, 0.99), and those in the slowly increased (HR: 1.07; 95%CI: 1.03, 1.13) and sharply increased group (HR: 1.12; 95%CI: 1. 02, 1.23) had elevated risks of stroke. Among participants with overweight, the conclusions were similar to the normal weight group. We found that the HRs (95% CIs) of stroke in large decreased group and slightly decreased group were 0.84 (0.75, 0.95) and 0.94 (0.89, 0.99) compared with the stable weight group. However, moderate weight gain was not significantly associated with stroke (HR: 1.05; 95%CI: 0.97, 1.15). Finally, among subjects with obesity, the results suggested that only substantial weight loss, that is, weight reduction to the normal range, could reduce the risk of stroke (HR: 0.79; 95%CI: 0.64, 0.96).

**Table 2:** HRs and 95%CIs of stroke risks for each BMI trajectory group among elderly individuals.

BMI at baseline	Trajectory	Number of stroke and population	Model 1	Model 2	Model 3
Underweight	Stable	404/4297	1.00 (Ref)	1.00 (Ref)	1.00 (Ref)
	Slightly increased	382/3907	1.04 (0.91, 1.20)	1.03 (0.90, 1.18)	1.05 (0.91, 1.21)
	Large increased	93/771	1.31 (1.04, 1.64)	1.29 (1.03, 1.61)	1.22 (0.97, 1.55)
Normal weight	Stable	4111/35475	1.00 (Ref)	1.00 (Ref)	1.00 (Ref)
	Slightly decreased	1709/16404	0.90 (0.85, 0.95)	0.90 (0.85, 0.95)	0.93 (0.88, 0.99)
	Slightly increased	3403/26385	1.12 (1.07, 1.17)	1.11 (1.06, 1.16)	1.07 (1.03, 1.13)
	Large increased	559/4160	1.17 (1.07, 1.28)	1.16 (1.06, 1.26)	1.12 (1.02, 1.23)
Overweight	Stable	2677/19082	1.00 (Ref)	1.00 (Ref)	1.00 (Ref)
	Large decreased	337/3001	0.79 (0.71, 0.89)	0.80 (0.72, 0.90)	0.84 (0.75, 0.95)
	Slightly decreased	2557/19851	0.92 (0.87, 0.97)	0.92 (0.87, 0.97)	0.94 (0.89, 0.99)
	Moderate increased	706/4712	1.07 (0.99, 1.17)	1.07 (0.98, 1.16)	1.05 (0.97, 1.15)
Obese	Stable	658/4580	1.00 (Ref)	1.00 (Ref)	1.00 (Ref)
	Large decreased	125/1103	0.78 (0.64, 0.94)	0.79 (0.65, 0.96)	0.79 (0.64, 0.96)
	Slightly decreased	851/6120	0.97 (0.87, 1.07)	0.97 (0.87, 1.07)	0.97 (0.88, 1.08)
	Slightly increased	142/965	1.02 (0.85, 1.22)	1.02 (0.85, 1.22)	1.02 (0.85, 1.22)

Model 1 was unadjusted.

Model 2 was adjusted for sex and age.

Model 3 was adjusted for sex, age, marital status, smoking status, physical exercise, drinking status, total cholesterol, triglyceride, diabetes, hypertension, hyperlipidemia, coronary heart disease and family history of stroke.

### 3.4 Sensitivity analysis

To conduct a sensitivity analysis, we stratified the subjects by sex (male and female) and age (grouped by every 5-year age interval). After fully adjustment, a decrease in BMI was associated with a reduced risk of stroke in men. For women, the results were basically consistent with the general population (Supplementary Table 2). The results of analysis by age indicated that for 75 ~ 80 and ≥80 years old subgroups, no significant association had been observed between weight gain and the risk of stroke (Supplementary Table 3).

## 4 Discussion

In this cohort study, a total of 15 distinct BMI trajectories were identified among 150, 813 elderly individuals. The findings suggested that among individuals with normal-weight, overweight, and obesity, a reduced BMI was a protective factor against stroke. In subjects with normal-weight, an increased BMI appears to be a risk factor for stroke.

During the 10-year follow-up period, a total of 18, 714 (12.41%) stroke events were documented. Compared with other studies, the incidence rate in our study was higher than the 6.3 and 6.8% reported in two retirement-cohort studies from China and the United States (24, 25), and 8.3% in an Atherosclerosis Risk in Communities Study (26) but close to the 9.4% in another Cardiovascular Health Study (27). This may be attributed to the fact that the mean age in our study (71.36) was higher than that reported in the previous three studies (58.3, 61, and 63), but was comparable to the average age observed in the Cardiovascular Health Study (78.1). Additionally, this study focused on longitudinal BMI trajectories, and BMI variability itself is closely associated with metabolic abnormalities, insulin resistance, and increased cardiovascular risk. Previous studies have demonstrated that high variability in metabolic syndrome parameters is significantly associated with stroke risk (28). Therefore, the included population may possess higher cardiometabolic risk profiles, contributing to a higher stroke rate than that observed in the general elderly population. Furthermore, following a stroke event, patients and their families may exhibit heightened health awareness, leading to increased engagement in regular health check-ups and chronic disease management. This enhanced healthcare utilization may increase the likelihood of stroke survivors being captured in our study.

A total of 6 distinct types of BMI trajectories were identified among people with baseline BMI classifications in our study: stable, slightly increased, moderate increased, large increased, slightly decreased, and large decreased. These trajectory types were generally consistent with those identified in other large cohort studies involving people over 50 years of age (29–31). An “increase to decrease” trajectory was observed in The National Health and Nutrition Examination Survey (NHANES) study while this pattern was not evident in our study (31).

The relationship between obesity and stroke has been demonstrated, and epidemiological meta-analyses have found that overweight and obesity in young adulthood are associated with an increased risk of stroke (32). Existing evidence had shown that obesity is a risk factors for cerebrovascular disease (17). The major underlying mechanisms by which obesity contributes to stroke include hypertension and atrial fibrillation, as well as a pro-thrombotic and pro-inflammatory state (2, 17, 33). Therefore, it is essential to develop more effective methods to monitor the dynamic changes in BMI in order to achieve a more precise assessment. We observed that an increased BMI was associated with an elevated risk of stroke, even when BMI was within the normal range. The Japan Public Health Center-Based Prospective (JPHC) Study observed that weight gaining ≥5 kg over 5 years was associated with a higher risk of total stroke in women (HR: 1.61; 95% CI: 1.36, 1.92) (34). The Oslo Ischemia Study revealed similar findings in men. Researchers estimated weight changes during early-life and mid-life among 2, 014 healthy men and found that weight gain of 5.0 to 9.9 kg was associated with a significantly higher risk of stroke compared to men who experienced no or minimal weight change (HR: 1. 46; 95% CI: 1. 09, 1.95) (35). Based on data from the China Health and Retirement Longitudinal Study, researchers also found that among people with type 2 diabetes, a one-unit dynamic increase in BMI over 2 years was associated with a 15.3% excess risk of incident stroke in men, while the association was insignificant in women (36). However, in our sensitivity analysis of males and females, after fully adjustment, the association between weight gain and stroke was not significant in men but notable in women. This association was more pronounced in women. The underlying mechanisms are not merely attributable to longer life expectancy, but also to sex-specific differences in adipose tissue distribution, hormonal fluctuations, and metabolic profiles. Under the regulation of estrogen, women present a distinct abdominal fat deposition pattern compared with men (37). Accordingly, elevated waist circumference is more strongly associated with stroke risk in females. Furthermore, the rapid decline in estrogen during the perimenopausal period, together with a higher prevalence of metabolic syndrome and related metabolic disorders, further increases the susceptibility to cardiovascular and cerebrovascular events.

The study further indicated that among individuals with overweight and obesity, a reduction in BMI to the normal range was associated with a decreased risk of stroke. In the normal weight population, even a modest decrease in weight was also associated with a reduced risk of stroke, suggesting that maintaining BMI may have a protective effect against stroke. These findings provide strategies and evidence for the primary prevention of stroke in the elderly. The American Heart Association/American Stroke Association has made screening for and recognizing obesity and adiposity an important first step in the evaluation of stroke risk in primary care (14). Consequently, weight management is essential for stroke prevention in elderly people, irrespective of baseline BMI classification.

The present study had several notable strengths. Initially, GBTM was used to model the trajectory of BMI over a 10-year period in adults 65 years of age and older. A sufficiently long follow-up duration was crucial to ensure the accurate identification of trends in BMI. Additionally, this study involved more than 150, 000 people, which enhances the representativeness of our findings with respect to the elderly population. However, this study also had several limitations. First, this study employed a retrospective design based on real-world data. By relying on historical records, retrospective cohort studies may limit the ability to comprehensively identify and control for all potential confounders. Consequently, unmeasured or residual confounding may introduce bias, potentially compromising the validity and reliability of the findings. Second, as depicted in Supplementary Table 4, the analytical population varied compared to the initial population, therefore, elderly individuals with higher life expectancy, such as a lower age of inclusion, a higher marital rate, lower levels of fasting plasma glucose (FPG), triglycerides (TG) and total cholesterol (TC), and a greater proportion of females, which may indeed influence our results and lead us to underestimate the relationship between BMI trajectory and stroke. Third, the present study utilized self-reported stroke diagnosis as the primary outcome, which carries the potential for reporting bias and overdiagnosis. Fourth, BMI variation represents a long-term trend, while stroke may have already occurred during the period of BMI change, which may introduce temporal ambiguity in the exposure-outcome relationship. Fifth, our findings related solely to changes in BMI, while it is weak to discriminate between fat and lean body mass and unable to account for different patterns of body composition and regional fat distribution. Future research should be extended to include more diverse populations and employ community-based random sampling to enhance representativeness. More precise models should be developed, and clinical event adjudication should be integrated with neuroimaging evidence to validate the findings.

## 5 Conclusion

In summary, during the 10-year follow-up period, among elderly individuals aged 65 and above compared with participants who maintained a stable weight, those with progressively decreasing weight trajectories exhibited a lower risk of stroke, whereas those with progressively increasing weight had a higher risk. Future studies should identify the biological pathways underlying these associations and replicate the findings in large cohort studies.

## Data Availability

The raw data supporting the conclusions of this article will be made available by the authors, without undue reservation.
