# Supplementary material for: Association of body mass index trajectories with incidence of stroke among elderly Chinese adults: a 10-year cohort study
Source: Front Nutr. 2026 Jun 10;13:1804187. doi: 10.3389/fnut.2026.1804187 (PMC13290690; doi:10.3389/fnut.2026.1804187)
Supplement: Supplementary file 1 [file Table_1.DOCX]

Supplementary Material

# Supplementary Tables

| **Supplementary table 1-1. Parameters in the process of BMI trajectory fitting among subjects with underweight (N=8975).** | | | | | |
| --- | --- | --- | --- | --- | --- |
| Groups numbers | Degrees of polynomial | BIC | Average posterior probabilities | Participants per class (%) | Sample size of each group |
| 1 | linear | -87721.3 | 0.95/0.89 | 74.65/25.35 | 6700/2275 |
|  | quadratic | -86468.0 | 0.96/0.91 | 74.32/25.68 | 6670/2305 |
|  | cubic | -86133.5 | 0.96/0.91 | 74.34/25.66 | 6672/2303 |
| 3 | linear | -85561.0 | 0.88/0.85/0.89 | 46.33/44.19/9.48 | 4158/3966/851 |
|  | quadratic | -83923.7 | 0.90/0.87/0.91 | 47.73/43.44/8.82 | 4284/3899/792 |
|  | **cubic** | **-83484.0** | **0.90/0.88/0.92** | **47.88/43.53/8.59** | **4297/3907/771** |
| 4 | linear | -84694.6 | 0.85/0.82/0.82/0.88 | 32.28/49.18/15.06/3.48 | 2897/4414/1352/312 |
|  | quadratic | -82924.2 | 0.86/0.84/0.85/0.91 | 28.94/48.66/18.46/3.94 | 2597/4367/1657/354 |
|  | cubic | -82427.3 | 0.87/0.84/0.86/0.92 | 30.06/48.56/18.01/3.38 | 2698/4358/1616/303 |

Abbreviations: BIC: Bayesian Information Criterion.

| **Supplementary table 1-2. Parameters in the process of BMI trajectory fitting among subjects with normal weight (N=82424).** | | | | | |
| --- | --- | --- | --- | --- | --- |
| Groups numbers | Degrees of polynomial | BIC | Average posterior probabilities | Participants per class (%) | Sample size of each group |
| 2 | linear | -832660.1 | 0.93/0.93 | 50.7/49.3 | 41790/40634 |
|  | quadratic | -830500.4 | 0.93/0.93 | 51.24/48.76 | 42233/40191 |
|  | cubic | -829812.2 | 0.93/0.93 | 51.48/48.52 | 42433/39991 |
| 3 | linear | -807978.8 | 0.91/0.89/0.89 | 31.34/53.58/15.08 | 25832/44160/12432 |
|  | quadratic | -804278.2 | 0.92/0.90/0.89 | 31.68/53.88/14.44 | 26113/44410/11901 |
|  | cubic | -803108.0 | 0.92/0.90/0.90 | 31.76/54.01/14.23 | 26181/44515/11728 |
| 4 | linear | -797414.8 | 0.89/0.84/0.85/0.89 | 19.55/42.11/32.83/5.52 | 16111/34708/27058/4547 |
|  | quadratic | -792648.4 | 0.89/0.85/0.87/0.90 | 19.92/43/32.05/5.04 | 16418/35440/26413/4153 |
|  | **cubic** | **-791179.2** | **0.89/0.85/0.87/0.91** | **19.9/43.04/32.01/5.05** | **16404/35475/26385/4160** |
| 5 | linear | -792369.8 | 0.86/0.81/0.81/0.83/0.89 | 12.81/32.98/38.16/13.95/2.09 | 10562/27187/31454/11501/1720 |
|  | quadratic | -787244.2 | 0.87/0.82/0.82/0.84/0.91 | 12.09/32.01/38.28/15.26/2.36 | 9963/26385/31554/12576/1946 |
|  | cubic | -785653.1 | 0.87/0.82/0.82/0.85/0.91 | 12.11/32.18/38.31/15.08/2.31 | 9979/26528/31579/12433/1905 |

Abbreviations: BIC: Bayesian Information Criterion.

| **Supplementary table 1-3. Parameters in the process of BMI trajectory fitting among subjects with overweight (N=46646).** | | | | | |
| --- | --- | --- | --- | --- | --- |
| Groups numbers | Degrees of polynomial | BIC | Average posterior probabilities | Participants per class (%) | Sample size of each group |
| 2 | linear | -473742.8 | 0.92/0.91 | 55.82/44.18 | 26036/20610 |
|  | quadratic | -472728.2 | 0.92/0.91 | 55.37/44.63 | 25827/20819 |
|  | cubic | -472319.8 | 0.93/0.92 | 55.32/44.68 | 25803/20843 |
| 3 | linear | -462699.1 | 0.86/0.87/0.89 | 22.08/57.35/20.57 | 10299/26753/9594 |
|  | quadratic | -461075.6 | 0.87/0.88/0.89 | 21.77/57.77/20.45 | 10156/26949/9541 |
|  | cubic | -460469.1 | 0.88/0.88/0.89 | 21.28/58.04/20.68 | 9925/27075/9646 |
| 4 | linear | -457407.2 | 0.86/0.84/0.84/0.87 | 7.3/43.2/39.67/9.83 | 3405/20149/18505/4587 |
|  | quadratic | -455339.7 | 0.87/0.86/0.85/0.88 | 6.53/42.4/40.93/10.14 | 3045/19778/19094/4729 |
|  | **cubic** | **-454586.2** | **0.88/0.86/0.85/0.88** | **6.43/42.56/40.91/10.1** | **3001/19851/19082/4712** |
| 5 | linear | -455067.5 | 0.86/0.8/0.79/0.81/0.86 | 3.29/23.98/42.82/25.03/4.88 | 1534/11186/19972/11677/2277 |
|  | quadratic | -452784.8 | 0.88/0.82/0.8/0.82/0.87 | 3.01/23.57/43.21/25.40/4.81 | 1404/10994/20156/11847/2245 |
|  | cubic | -451963.3 | 0.88/0.82/0.8/0.83/0.87 | 2.98/23.57/43.18/25.44/4.84 | 1388/10993/20144/11865/2256 |

Abbreviations: BIC: Bayesian Information Criterion.

| **Supplementary table 1-4. Parameters in the process of BMI trajectory fitting among subjects with obesity (N=12768).** | | | | | |
| --- | --- | --- | --- | --- | --- |
| Groups numbers | Degrees of polynomial | BIC | Average posterior probabilities | Participants per class (%) | Sample size of each group |
| 2 | linear | -144894.7 | 0.93/0.90 | 67.55/32.45 | 8625/4143 |
|  | quadratic | -144465.5 | 0.93/0.90 | 66.41/33.59 | 8479/4289 |
|  | cubic | -144288.0 | 0.93/0.9 | 66.03/33.97 | 8431/4337 |
| 3 | linear | -141498.8 | 0.86/0.89/0.9 | 25.27/60.74/14.00 | 3226/7755/1787 |
|  | quadratic | -140797.3 | 0.87/0.9/0.9 | 23.57/61.63/14.79 | 3010/7869/1889 |
|  | cubic | -140523.5 | 0.88/0.91/0.9 | 22.68/62.10/15.22 | 2896/7929/1943 |
| 4 | linear | -139814.7 | 0.86/0.85/0.84/0.9 | 8.8/48.46/35.42/7.32 | 1123/6188/4523/934 |
|  | quadratic | -138981.0 | 0.88/0.86/0.85/0.91 | 8.32/47.7/36.3/7.68 | 1062/6090/4635/981 |
|  | **cubic** | **-138685.3** | **0.88/0.87/0.85/0.91** | **8.64/47.93/35.87/7.56** | **1103/6120/4580/965** |
| 5 | linear | -139039.8 | 0.85/0.79/0.8/0.84/0.9 | 5.23/27.15/45.32/18.73/3.56 | 668/3467/5787/2392/454 |
|  | quadratic | -138139.2 | 0.87/0.81/0.82/0.85/0.9 | 4.8/26.83/45.9/18.8/3.66 | 613/3426/5861/2401/467 |
|  | cubic | -137841.0 | 0.88/0.82/0.82/0.85/0.9 | 4.96/27.35/45.54/18.6/3.55 | 633/3492/5815/2375/453 |

Abbreviations: BIC: Bayesian Information Criterion.

| **Supplementary table 2-1. HRs and 95%CIs of stroke risks for each BMI trajectory group among male elderly individuals.** | | | | | |
| --- | --- | --- | --- | --- | --- |
| BMI at Baseline | Trajectory | Number of Stroke and population | Model 1 | Model 2 | Model 3 |
|  |  |  |  |  |  |
| Underweight | Stable | 179/1984 | 1.00 (Ref) | 1.00 (Ref) | 1.00 (Ref) |
|  | Slightly increased | 162/1766 | 1.02 (0.82, 1.26) | 1.01 (0.82, 1.25) | 1.01 (0.81, 1.25) |
|  | Large increased | 41/322 | **1.45 (1.04, 2.04)** | **1.44 (1.03, 2.02)** | 1.25 (0.87, 1.80) |
| Normal weight | Stable | 1917/16906 | 1.00 (Ref) | 1.00 (Ref) | 1.00 (Ref) |
|  | Slightly decreased | 810/8148 | **0.87 (0.80, 0.95)** | **0.87 (0.80, 0.95)** | **0.89 (0.82, 0.97)** |
|  | Slightly increased | 1573/12574 | **1.11 (1.04, 1.18)** | **1.11 (1.04, 1.18)** | 1.06 (0.99, 1.13) |
|  | Large increased | 227/1761 | **1.14 (1.00, 1.31)** | **1.14 (1.00, 1.31)** | 1.06 (0.92, 1.23) |
| Overweight | Stable | 1208/8894 | 1.00 (Ref) | 1.00 (Ref) | 1.00 (Ref) |
|  | Large decreased | 140/1274 | **0.80 (0.67, 0.96)** | **0.80 (0.67, 0.95)** | **0.83 (0.69, 0.99)** |
|  | Slightly decreased | 1142/9245 | **0.91 (0.84, 0.98)** | **0.91 (0.84, 0.98)** | **0.92 (0.85, 1.00)** |
|  | Moderate increased | 269/1944 | 1.02 (0.89, 1.16) | 1.02 (0.89, 1.16) | 1.02 (0.89, 1.16) |
| Obese | Stable | 222/1663 | 1.00 (Ref) | 1.00 (Ref) | 1.00 (Ref) |
|  | Large decreased | 48/386 | 0.93 (0.68, 1.28) | 0.92 (0.68, 1.26) | 0.91 (0.66, 1.27) |
|  | Slightly decreased | 371/2465 | 1.14 (0.96, 1.34) | 1.13 (0.96, 1.34) | 1.15 (0.97, 1.36) |
|  | Slightly increased | 31/239 | 0.97 (0.67, 1.41) | 0.98 (0.67, 1.42) | 1.00 (0.69, 1.46) |

Model 1 was unadjusted.

Model 2 was adjusted for age.

Model 3 was adjusted for sex, age, marital status, smoking status, physical exercise, drinking status, total cholesterol, triglyceride, diabetes, hypertension, hyperlipidemia, coronary heart disease and family history of stroke.

| **Supplementary table 2-2. HRs and 95%CIs of stroke risks for each BMI trajectory group among female elderly individuals.** | | | | | |
| --- | --- | --- | --- | --- | --- |
| BMI at Baseline | Trajectory | Number of Stroke and population | Model 1 | Model 2 | Model 3 |
|  |  |  |  |  |  |
| Underweight | Stable | 225/2313 | 1.00 (Ref) | 1.00 (Ref) | 1.00 (Ref) |
|  | Slightly increased | 220/2141 | 1.06 (0.88, 1.27) | 1.04 (0.86, 1.25) | 1.08 (0.89, 1.31) |
|  | Large increased | 52/449 | 1.20 (0.89, 1.62) | 1.18 (0.87, 1.60) | 1.22 (0.89, 1.66) |
| Normal weight | Stable | 2194/18569 | 1.00 (Ref) | 1.00 (Ref) | 1.00 (Ref) |
|  | Slightly decreased | 899/8256 | **0.92 (0.85, 0.99)** | 0.93 (0.86, 1.01) | 0.97 (0.90, 1.05) |
|  | Slightly increased | 1830/13811 | **1.13 (1.06, 1.2)** | **1.11 (1.05, 1.19)** | **1.09 (1.02, 1.16)** |
|  | Large increased | 332/2399 | **1.19 (1.06, 1.33)** | **1.17 (1.04, 1.31)** | **1.17 (1.04, 1.31)** |
| Overweight | Stable | 1469/10188 | 1.00 (Ref) | 1.00 (Ref) | 1.00 (Ref) |
|  | Large decreased | 197/1727 | **0.79 (0.68, 0.91)** | **0.81 (0.70, 0.94)** | **0.86 (0.74, 1.00)** |
|  | Slightly decreased | 1415/10606 | **0.92 (0.86, 0.99)** | 0.94 (0.87, 1.01) | 0.95 (0.89, 1.03) |
|  | Moderate increased | 437/2768 | 1.10 (0.99, 1.23) | 1.10 (0.99, 1.22) | 1.08 (0.97, 1.20) |
| Obese | Stable | 436/2917 | 1.00 (Ref) | 1.00 (Ref) | 1.00 (Ref) |
|  | Large decreased | 77/717 | **0.70 (0.55, 0.90)** | **0.73 (0.57, 0.93)** | **0.73 (0.57, 0.94)** |
|  | Slightly decreased | 480/3655 | **0.87 (0.76, 0.99)** | 0.88 (0.77, 1.00) | 0.89 (0.78, 1.01) |
|  | Slightly increased | 111/726 | 1.02 (0.83, 1.25) | 1.01 (0.82, 1.25) | 1 .00(0.81, 1.24) |

Model 1 was unadjusted.

Model 2 was adjusted for age.

Model 3 was adjusted for sex, age, marital status, smoking status, physical exercise, drinking status, total cholesterol, triglyceride, diabetes, hypertension, hyperlipidemia, coronary heart disease and family history of stroke.

| **Supplementary table 3-1. HRs and 95%CIs of stroke risks for each BMI trajectory group among participants aged 65-70 years.** | | | | | |  |
| --- | --- | --- | --- | --- | --- | --- |
| BMI at Baseline | Trajectory | Number of Stroke and population | Model 1 | Model 2 | Model 3 |  |
|  |  |  |  |  |  |  |
| Underweight | Stable | 135/1436 | 1.00 (Ref) | 1.00 (Ref) | 1.00 (Ref) |  |
|  | Slightly increased | 152/1442 | 1.13 (0.90, 1.42) | 1.13 (0.89, 1.42) | 1.15 (0.91, 1.46) |  |
|  | Large increased | 40/311 | 1.41 (0.99, 2.00) | 1.39 (0.98, 1.98) | 1.31 (0.90, 1.90) |  |
| Normal weight | Slightly decreased | 641/6112 | **0.90 (0.82, 0.98)** | **0.90 (0.82, 0.99)** | 0.93 (0.85, 1.02) |  |
|  | Stable | 1711/14693 | 1.00 (Ref) | 1.00 (Ref) | 1.00 (Ref) |  |
|  | Slightly increased | 1515/11953 | **1.09 (1.02, 1.17)** | **1.09 (1.02, 1.17)** | 1.05 (0.98, 1.12) |  |
|  | Large increased | 280/1982 | **1.23 (1.08, 1.39)** | **1.22 (1.08, 1.38)** | **1.16 (1.02, 1.32)** |  |
| Overweight | Stable | 1332/9107 | 1.00 (Ref) | 1.00 (Ref) | 1.00 (Ref) |  |
|  | Large decreased | 149/1128 | **0.90 (0.76, 1.06)** | **0.89 (0.75, 1.06)** | **0.92 (0.78, 1.10)** |  |
|  | Slightly decreased | 1120/8684 | **0.88 (0.81, 0.95)** | **0.88 (0.81, 0.95)** | **0.91 (0.84, 0.98)** |  |
|  | Moderate increased | 365/2346 | 1.07 (0.95, 1.20) | 1.06 (0.94, 1.19) | 1.04 (0.92, 1.16) |  |
| Obese | Stable | 328/2272 | 1.00 (Ref) | 1.00 (Ref) | 1.00 (Ref) |  |
|  | Large decreased | 41/414 | **0.68 (0.49, 0.94)** | **0.68 (0.49, 0.93)** | **0.68 (0.49, 0.95)** |  |
|  | Slightly decreased | 389/2834 | 0.95 (0.82, 1.1) | 0.95 (0.82, 1.10) | 0.97 (0.84, 1.13) |  |
|  | Slightly increased | 86/519 | 1.16 (0.91, 1.47) | 1.15 (0.91, 1.46) | 1.16 (0.91, 1.47) |  |

Model 1 was unadjusted.

Model 2 was adjusted for sex and age.

Model 3 was adjusted for sex, age, marital status, smoking status, physical exercise, drinking status, total cholesterol, triglyceride, diabetes, hypertension, hyperlipidemia, coronary heart disease and family history of stroke.

| **Supplementary table 3-2. HRs and 95%CIs of stroke risks for each BMI trajectory group among participants aged 70-75 years.** | | | | | |  |
| --- | --- | --- | --- | --- | --- | --- |
| BMI at Baseline | Trajectory | Number of Stroke and population | Model 1 | Model 2 | Model 3 |  |
|  |  |  |  |  |  |  |
| Underweight | Stable | 150/1449 | 1.00 (Ref) | 1.00 (Ref) | 1.00 (Ref) |  |
|  | Slightly increased | 145/1320 | 1.06 (0.85, 1.34) | 1.06 (0.85, 1.34) | 1.08 (0.85, 1.36) |  |
|  | Large increased | 32/242 | 1.31 (0.89, 1.92) | 1.30 (0.89, 1.91) | 1.25 (0.84, 1.84) |  |
| Normal weight | Stable | 1451/11685 | 1.00 (Ref) | 1.00 (Ref) | 1.00 (Ref) |  |
|  | Slightly decreased | 620/5423 | **0.92 (0.83, 1.01)** | **0.92 (0.83, 1.01)** | **0.93 (0.85, 1.03)** |  |
|  | Slightly increased | 1253/8685 | **1.17 (1.09, 1.27)** | **1.17 (1.09, 1.27)** | **1.14 (1.05, 1.23)** |  |
|  | Large increased | 190/1410 | 1.09 (0.94, 1.27) | 1.09 (0.94, 1.27) | 1.04 (0.89, 1.22) |  |
| Overweight | Stable | 885/6354 | 1.00 (Ref) | 1.00 (Ref) | 1.00 (Ref) |  |
|  | Large decreased | 103/946 | **0.78 (0.63, 0.95)** | **0.77 (0.63, 0.95)** | 0.82 (0.66, 1.01) |  |
|  | Slightly decreased | 886/6561 | 0.97 (0.88, 1.06) | 0.97 (0.88, 1.06) | 0.98 (0.89, 1.08) |  |
|  | Moderate increased | 253/1583 | 1.16 (1.01, 1.34) | 1.16 (1.01, 1.33) | 1.16 (1.01, 1.34) |  |
| Obese | Stable | 229/1490 | 1.00 (Ref) | 1.00 (Ref) | 1.00 (Ref) |  |
|  | Large decreased | 52/358 | 0.94 (0.69, 1.26) | 0.93 (0.69, 1.26) | 0.95 (0.70, 1.29) |  |
|  | Slightly decreased | 288/1969 | 0.94 (0.79, 1.12) | 0.94 (0.79, 1.12) | 0.95 (0.80, 1.13) |  |
|  | Slightly increased | 40/307 | 0.83 (0.60, 1.17) | 0.83 (0.59, 1.16) | 0.84 (0.6, 1.18) |  |

Model 1 was unadjusted.

Model 2 was adjusted for sex and age.

Model 3 was adjusted for sex, age, marital status, smoking status, physical exercise, drinking status, total cholesterol, triglyceride, diabetes, hypertension, hyperlipidemia, coronary heart disease and family history of stroke.

| **Supplementary table 3-3. HRs and 95%CIs of stroke risks for each BMI trajectory group among participants aged 75-80 years.** | | | | | |  |
| --- | --- | --- | --- | --- | --- | --- |
| BMI at Baseline | Trajectory | Number of Stroke and population | Model 1 | Model 2 | Model 3 |  |
|  |  |  |  |  |  |  |
| Underweight | Stable | 96/985 | 1.00 (Ref) | 1.00 (Ref) | 1.00 (Ref) |  |
|  | Slightly increased | 61/786 | 0.79 (0.57, 1.09) | 0.78 (0.57, 1.08) | 0.82 (0.59, 1.14) |  |
|  | Large increased | 14/135 | 1.06 (0.60, 1.86) | 1.05 (0.60, 1.84) | 1.12 (0.63, 1.99) |  |
| Normal weight | Stable | 744/6586 | 1.00 (Ref) | 1.00 (Ref) | 1.00 (Ref) |  |
|  | Slightly decreased | 346/3476 | **0.87 (0.77, 0.99)** | **0.87 (0.77, 0.99)** | 0.91 (0.80, 1.03) |  |
|  | Slightly increased | 498/4255 | 1.04 (0.93, 1.16) | 1.03 (0.92, 1.16) | 1 (0.89, 1.12) |  |
|  | Large increased | 71/580 | 1.09 (0.86, 1.39) | 1.09 (0.86, 1.39) | 1.14 (0.89, 1.45) |  |
| Overweight | Stable | 364/2805 | 1.00 (Ref) | 1.00 (Ref) | 1.00 (Ref) |  |
|  | Large decreased | 67/645 | 0.80 (0.61, 1.03) | 0.80 (0.62, 1.04) | 0.86 (0.66, 1.12) |  |
|  | Slightly decreased | 414/3390 | 0.94 (0.82, 1.08) | 0.94 (0.82, 1.09) | 0.95 (0.82, 1.09) |  |
|  | Moderate increased | 71/619 | 0.88 (0.68, 1.13) | 0.88 (0.68, 1.14) | 0.86 (0.66, 1.12) |  |
| Obese | Stable | 90/659 | 1.00 (Ref) | 1.00 (Ref) | 1.00 (Ref) |  |
|  | Large decreased | 22/223 | 0.71 (0.45, 1.13) | 0.73 (0.46, 1.16) | 0.67 (0.41, 1.10) |  |
|  | Slightly decreased | 140/1003 | 1.02 (0.78, 1.33) | 1.03 (0.79, 1.34) | 1.05 (0.80, 1.37) |  |
|  | Slightly increased | 13/112 | 0.82 (0.46, 1.47) | 0.88 (0.49, 1.58) | 0.89 (0.5, 1.60) |  |

Model 1 was unadjusted.

Model 2 was adjusted for sex and age.

Model 3 was adjusted for sex, age, marital status, smoking status, physical exercise, drinking status, total cholesterol, triglyceride, diabetes, hypertension, hyperlipidemia, coronary heart disease and family history of stroke.

| **Supplementary table 3-4. HRs and 95%CIs of stroke risks for each BMI trajectory group among participants aged 80 years and older.** | | | | | |  |
| --- | --- | --- | --- | --- | --- | --- |
| BMI at Baseline | Trajectory | Number of Stroke and population | Model 1 | Model 2 | Model 3 |  |
|  |  |  |  |  |  |  |
| Underweight | Stable | 23/427 | 1.00 (Ref) | 1.00 (Ref) | 1.00 (Ref) |  |
|  | Slightly increased | 24/359 | 1.25 (0.71, 2.22) | 1.27 (0.72, 2.25) | 1.29 (0.70, 2.37) |  |
|  | Large increased | 7/83 | 1.59 (0.68, 3.71) | 1.64 (0.70, 3.82) | 1.32 (0.49, 3.53) |  |
| Normal weight | Stable | 205/2511 | 1.00 (Ref) | 1.00 (Ref) | 1.00 (Ref) |  |
|  | Slightly decreased | 102/1393 | **0.55 (0.34, 0.91)** | **0.56 (0.35, 0.92)** | **0.60 (0.37, 0.98)** |  |
|  | Slightly increased | 137/1492 | 1.04 (0.80, 1.35) | 1.03 (0.79, 1.34) | 1.01 (0.77, 1.31) |  |
|  | Large increased | 18/188 | 0.91 (0.55, 1.51) | 0.91 (0.55, 1.51) | 0.84 (0.50, 1.42) |  |
| Overweight | Stable | 96/816 | 1.00 (Ref) | 1.00 (Ref) | 1.00 (Ref) |  |
|  | Large decreased | 18/282 | **0.53 (0.32, 0.88)** | **0.55 (0.33, 0.91)** | **0.60 (0.36, 0.99)** |  |
|  | Slightly decreased | 137/1216 | 0.96 (0.74, 1.24) | 0.97 (0.75, 1.26) | 0.99 (0.76, 1.30) |  |
|  | Moderate increased | 17/164 | 0.87 (0.52, 1.46) | 0.88 (0.53, 1.48) | 0.84 (0.49, 1.42) |  |
| Obese | Stable | 11/159 | 1.00 (Ref) | 1.00 (Ref) | 1.00 (Ref) |  |
|  | Large decreased | 10/108 | 1.40 (0.59, 3.30) | 1.40 (0.59, 3.30) | 1.40 (0.57, 3.44) |  |
|  | Slightly decreased | 34/314 | 1.62 (0.82, 3.19) | 1.62 (0.82, 3.19) | 1.56 (0.78, 3.11) |  |
|  | Slightly increased | 3/27 | 1.68 (0.47, 6.04) | 1.68 (0.47, 6.04) | 1.85 (0.50, 6.77) |  |

Model 1 was unadjusted.

Model 2 was adjusted for sex and age.

Model 3 was adjusted for sex, age, marital status, smoking status, physical exercise, drinking status, total cholesterol, triglyceride, diabetes, hypertension, hyperlipidemia, coronary heart disease and family history of stroke.

**Supplementary Table 4 Comparison between the Included Population and the Excluded Population**

| **Variables** | **Included Population** | **Excluded Population** |  |
| --- | --- | --- | --- |
| Number | 150, 813 | 222, 526 |  |
| Age, mean(SD),years | 71.36±4.61 | 74.19±6.10 | t=155.70, P<0.0001 |
| Female (%) | 53.87 | 52.98 | 61.94, P<0.0001 |
| Married (%) | 76.91 | 69.98 | 2083.33, P<0.0001 |
| Exercise (%) | 28.58 | 38.39 | 205.86, P<0.0001 |
|  |  |  |  |
| Smoking (%) | 24.35 | 15.40 | 179.35, P<0.0001 |
| FPG, mean(SD),mmol/L | 5.44(1.37) | 5.63(1.37) | t=62.10, P<0.0001 |
| TG, mean(SD),mmol/L | 1.40(0.92) | 1.41(0.91) | t=3.48, P<0.0001 |
| TC, mean(SD),mmol/L | 4.91(1.00) | 4.92(1.07) | t=4.11, P<0.0001 |
